# The intersection of obesity and (long) COVID-19: Hypoxia, thrombotic inflammation, and vascular endothelial injury

**DOI:** 10.3389/fcvm.2023.1062491

**Published:** 2023-02-07

**Authors:** Mengqi Xiang, Xiaoming Wu, Haijiao Jing, Valerie A. Novakovic, Jialan Shi

**Affiliations:** ^1^Department of Hematology, The First Affiliated Hospital of Harbin Medical University, Harbin Medical University, Harbin, China; ^2^Department of Research, Veterans Affairs Boston Healthcare System and Harvard Medical School, Boston, MA, United States; ^3^Department of Medical Oncology, Dana-Farber Cancer Institute and Harvard Medical School, Boston, MA, United States

**Keywords:** COVID-19, long COVID, hypoxia, thrombotic inflammation, vascular endothelial injury

## Abstract

The role of hypoxia, vascular endothelial injury, and thrombotic inflammation in worsening COVID-19 symptoms has been generally recognized. Damaged vascular endothelium plays a crucial role in forming *in situ* thrombosis, pulmonary dysfunction, and hypoxemia. Thrombotic inflammation can further aggravate local vascular endothelial injury and affect ventilation and blood flow ratio. According to the results of many studies, obesity is an independent risk factor for a variety of severe respiratory diseases and contributes to high mechanical ventilation rate, high mortality, and slow recovery in COVID-19 patients. This review will explore the mechanisms by which obesity may aggravate the acute phase of COVID-19 and delay long COVID recovery by affecting hypoxia, vascular endothelial injury, and thrombotic inflammation. A systematic search of PubMed database was conducted for papers published since January 2020, using the medical subject headings of “COVID-19” and “long COVID” combined with the following keywords: “obesity,” “thrombosis,” “endothelial injury,” “inflammation,” “hypoxia,” “treatment,” and “anticoagulation.” In patients with obesity, the accumulation of central fat restricts the expansion of alveoli, exacerbating the pulmonary dysfunction caused by SARS-CoV-2 invasion, inflammatory damage, and lung edema. Abnormal fat secretion and immune impairment further aggravate the original tissue damage and inflammation diffusion. Obesity weakens baseline vascular endothelium function leading to an early injury and pre-thrombotic state after infection. Enhanced procoagulant activity and microthrombi promote early obstruction of the vascular. Obesity also prolongs the duration of symptoms and increases the risk of sequelae after hospital discharge. Persistent viral presence, long-term inflammation, microclots, and hypoxia may contribute to the development of persistent symptoms, suggesting that patients with obesity are uniquely susceptible to long COVID. Early interventions, including supplemental oxygen, comprehensive antithrombotic therapy, and anti-inflammatory drugs, show effectiveness in many studies in the prevention of serious hypoxia, thromboembolic events, and systemic inflammation, and are therefore recommended to reduce intensive care unit admission, mortality, and sequelae.

## Introduction

As we all know, obesity is an independent risk factor for severe or lethal complications of many diseases. Features such as weight load, low-grade inflammation, neuroendocrine factors, metabolic abnormalities, and nursing difficulties all play a role (1–4). The mechanical action of central fat affects the compliance of the respiratory system. Patients with obesity have higher levels of proinflammatory cytokines and inflammatory cells infiltrating adipose tissue, along with leptin resistance and low levels of anti-inflammatory adiponectin, which can modulate immune responses, affecting tissues and organs throughout the body. Additionally, obesity is often accompanied by diseases such as insulin resistance, abnormal lipid metabolism, high blood pressure, fatty liver, and coronary heart disease. These are independent risk factors in many diseases and may have additive effects. In the influenza A (H1N1) pandemic, the delayed antiviral response in obese patients exacerbated disease and increased mortality, while prolonged influenza A shedding and chronic inflammation contributed to poor recovery (3, 4). Despite recent worldwide efforts to study the cross-population of coronavirus disease 2019 (COVID-19) and obesity, little is known about how obesity adversely affects COVID-19 symptoms and long COVID sequelae (5–13).

A large number of cohort and case-control studies have shown that high body mass index (BMI) is a risk factor for increased disease severity and mortality in COVID-19 patients. This is primarily measured as increased prevalence of severe and critical illness, hospitalization rate, mechanical ventilation rate, intensive care unit (ICU) hospitalization rate, and in-hospital mortality (Table 1). In our pooled study, compared with normal-weight individuals, patients with a high BMI had a 1.35-fold increased risk of severe illness and a 2.35-fold increased risk of critical illness (14, 15). Data published by the American Heart Association, which included clinical information of 7,606 confirmed patients, showed that 61% of hospitalized patients were overweight while only 28% were normal weight (9). Individuals who were overweight or obese had a higher risk of invasive mechanical ventilation (IMV), with an adjusted risk ratio (aRR) of 1.12 [95% confidence interval (CI) 1.05–1.19] and 2.08 (95% CI 1.89–2.29), respectively. There was also a strong correlation between increased BMI and death (16). The risk of ICU admission in individuals with obesity ranged from 1.06 to 1.89 (6, 14, 16). In long COVID, higher BMI was associated with longer symptom duration and delayed recovery. Palaiodimos et al. showed that the in-hospital mortality rate of patients with BMI ≥ 35 kg/m^2^ was approximately twice that of patients with BMI between 25 and 34 kg/m^2^ (34.8 vs. 17.2%) (17). In another study, aRR for patients with a BMI of 30–34.9 kg/m^2^ was 1.08 (95% CI 1.02–1.14) and 1.61 (95% CI 1.47–1.76) for patients with BMI ≥ 45 kg/m^2^ compared to patients of normal weight (16). The obesity rate of non-survivors and survivors was 27.1 and 13.5%, respectively (18). The adjusted hazard ratio (aHR) of prolonged symptoms in patients with BMI 25–30 kg/m^2^ and those with BMI > 30 kg/m^2^ were 1.07 (95% CI 1.04–1.10) and 1.10 (95% CI 1.07–1.14), respectively (11). Individuals with a BMI of >30 kg/m^2^ were less likely to recover within 1 year after discharge (12, 13).

**TABLE 1:** Studies reporting on the outcomes of obese patients with COVID-19.

References	Study population	BMI (kg/m^2^)	Outcomes
Simonnet et al. (140)	124 patients admitted to ICU	30 < BMI ≤ 35 (47.6%) BMI > 35 (28.2%)	The proportion of patients requiring mechanical ventilation (*p* < 0.01): BMI > 35 (85.7%) 30 < BMI ≤ 35 (75%) 25 < BMI ≤ 30 (60.4%) BMI < 25 (47.1%)
Lighter et al. (141)	3,615 SARS-CoV-2 positive patients	30 ≤ BMI < 35 (21%) BMI ≥ 35 (16%)	Critical disease: BMI of 30–34 is 1.8 times more than normal. BMI > 35 is 3.6 times more than normal.
Cai et al. (142)	383 confirmed inpatients	18.5 ≤ BMI ≤ 23.9 (53.1%) 24.0 ≤ BMI ≤ 27.9 (32.0%) BMI ≥ 28 (10.7%)	Probability of developing severe cases: BMI: 18.5–23.9 (19.2%) BMI: 24.0–27.9 (29.3%) BMI ≥ 28 (39.0%) (*p* = 0.001)
Hamer et al. (7)	334,329 cases of samples 640 confirmed inpatients	BMI > 25 (66.6%)	Possibility of hospitalization compared to normal weight: BMI: 25–30 (OR 1.39) BMI: 30–35 (OR 1.70) BMI > 35 (OR 3.3)
Giacomelli et al. (18)	233 confirmed inpatients	BMI > 30 (16.3%)	Obesity rate among survivors (13.5%) Obesity rate among non-survivors (27.1%) Mortality of BMI > 30 (aHR 3.04)
Huang et al. (6)	A summary of 33 articles	Not reported	Univariate analysis of COVID-19 patients with obesity: Risk of hospitalization (OR 1.76, *p* = 0.003) Risk of ICU admission (OR 1.67, *p* < 0.001) Risk of death (OR 1.37, *p* = 0.014) Risk of IMV (OR 2.19, *p* < 0.001)
Palaiodimos et al. (17)	200 confirmed inpatients	BMI < 25 (19%) BMI: 25–34 (58%) BMI ≥ 35 (23%)	In-hospital mortality rate: BMI < 25 (31.6%) BMI: 25–34 (17.2%) BMI ≥ 35 (34.8%) Intubation rate: BMI < 25 (18.4%) BMI: 25–34 (16.4%) BMI ≥ 35 (34.8%)
Kass et al. (143)	265 patients admitted to ICU	BMI < 26 (25%) BMI > 34.7 (25%)	Younger individuals admitted to hospital were more likely to be obese.
Bhatraju et al. (144)	24 patients admitted to ICU	BMI: 18–25 (3) BMI: 25–30 (7) BMI > 30 (13)	BMI > 30 (85% required mechanical ventilation and 62% died) BMI < 30 (64% required mechanical ventilation and 36% died)
Petrilli et al. (145)	4,103 confirmed patients	BMI > 30 (26.8%)	Rate of obesity among hospitalized patients: 39.8% BMI > 40 kg/m^2^ is the biggest risk factor for hospitalization (OR 6.2)
Goyal et al. (146)	393 confirmed inpatients	BMI > 30 (35.8%)	Obesity accounted for 43.3% of patients requiring invasive ventilation. Obesity accounted for 31.9% of non-invasive ventilation patients.
Caussy et al. (14)	340 patients with severe condition	BMI > 30 (25%)	After standardization of age and sex, compared to the average French person: The incidence of obesity in severe COVID-19 is 1.35 times higher (*p* = 0.0034). The prevalence of obesity in the ICU is 1.89 times higher (*p* = 0.0011).
Du et al. (15)	109,881 patients with COVID-19 in the meta-analysis	Not reported	The observational studies showed that patients with a BMI ≥ 30 kg/m^2^ were 2.35 times more likely to develop critical COVID-19 and had a 2.68-fold risk for mortality, compared with patients with a BMI < 30 kg/m^2^. Random-effects dose-response meta-analysis showed that the incidence of critical cases and mortality augmented by 9 and 6% for each 1 kg/m^2^ increase in BMI, respectively.
Tartof et al. (147)	6,916 patients with COVID-19	BMI: 18.5–24 (*n* = 1,240) BMI: 25–29 (*n* = 2,207) BMI: 30–39 (*n* = 2,537) BMI: 40–44 (*n* = 372) BMI ≥ 45 (*n* = 262)	Compared with patients with 18.5 ≤ BMI<24 kg/m^2^, those with BMI of 40–44 kg/m^2^ and greater than 45 kg/m^2^ had relative risks of 2.68 and 4.18, respectively.
Hendren et al. (9)	7,606 patients hospitalized with COVID-19	Underweight, BMI < 18.5 (*n* = 194) Normal, BMI: 18.5–24.9 (*n* = 1,793) Overweight, BMI: 25.0–29.9 (*n* = 2,308) Class I obesity, BMI: 30.0–34.9 (*n* = 1,623) Class II obesity, BMI: 35.0–39.9 (*n* = 846) Class III obesity, BMI ≥ 40.0 (*n* = 842)	Higher risks of in-hospital death or mechanical ventilation than normal weight group (18.5–24.9 kg/m^2^): Class I obesity, BMI 30.0–34.9 kg/m^2^ (OR 1.28) Class II obesity, BMI 35.0–39.9 kg/m^2^ (OR 1.57) Class III obesity, BMI ≥ 40.0 kg/m^2^ (OR 1.80)
Kompaniyets et al. (16)	148,494 patients with COVID-19	Underweight, BMI < 18.5 (*n* = 79,988, 2.5%) Healthy weight, BMI: 18.5–24.9 (*n* = 829,474, 25.6%) Overweight, BMI: 25–29.9 (*n* = 936,132, 28.9%) Obesity, BMI ≥ 30 (*n* = 1,397,055, 43.1%)	aRRs for hospitalization for patients with different BMI compared with healthy-weight cohort: Hospitalization: BMI 30–34.9 kg/m^2^: 1.07 (95% CI 1.05–1.09) BMI ≥ 45 kg/m^2^: 1.33 (95% CI 1.30–1.37) Death: BMI 30–34.9 kg/m^2^: 1.08 (95% CI 1.02–1.14) BMI ≥ 45 kg/m^2^: 1.61 (95% CI 1.47–1.76) ICU admission BMI 40–44.9 kg/m^2^: 1.06 (95% CI 1.03–1.10) BMI ≥ 45 kg/m^2^: 1.16 (95% CI 1.11–1.20) IMV: BMI 25–29.9 kg/m^2^: 1.12 (95% CI 1.05–1.19) BMI ≥ 45 kg/m2: 2.08 (95% CI 1.89–2.29)
Yamashita et al. (10)	1,236 patients with COVID-19	Mean body: 67.6 kg Mean BMI: 24.0 kg/m^2^	COVID-19 patients with VTE showed a higher body weight (81.6 vs. 64.0 kg, *p* = 0.005) and BMI (26.9 vs. 23.2 kg/m^2^, *P* = 0.04) compared with those without.
Thompson et al. (89)	1.1 million individuals with COVID-19 diagnostic codes in electronic healthcare records	Acute COVID-19 (*n* = 1,064,491) Not obese (*n* = 800,439) Obese I, BMI (30–34.9) (*n* = 151,782) Obese II (35–39.9) (*n* = 67,470) Obese III (40+) (*n* = 44,800) Long COVID (*n* = 4,189) Not obese (*n* = 2,694) Obese I (30–34.9) (*n* = 787) Obese II (35–39.9) (*n* = 411) Obese III (40+) (*n* = 297)	Overweight/obesity was associated with increased odds of symptoms lasting for 4+ weeks (OR 1.24, 95% CI 1.01–1.53) but not with symptoms lasting 12+ weeks specifically (OR 0.95, 95% CI 0.70–1.28).
Subramanian et al. (11)	486,149 adults with confirmed SARS-CoV-2 infection	BMI < 18.5 (*n* = 13,261, 2.7%) BMI: 18.5–25 (*n* = 148,295, 30.5%) BMI: 25–30 (*n* = 138,771, 28.5%) BMI > 30 (*n* = 121,943, 25.1%)	Compared with patients with normal BMI, patients with a BMI of 25–30 kg/m^2^ reported an aHR of 1.07 (95% CI 1.04–1.10) for prolonged symptoms and those with a BMI of >30 kg/m^2^ reported an aHR of 1.10 (95% CI 1.07–1.14).
PHOSP-COVID Collaborative Group (12)	924 post-COVID participants who had a 1-year visit	BMI < 30 kg/m^2^ (*n* = 349, 40.3%) BMI ≥ 30 kg/m^2^ (*n* = 517, 59.7%)	In multivariable analysis, BMI ≥ 30 kg/m^2^ (OR 0.50, 95% CI 0.34–0.74, *p* = 0.0007) was an independent factor associated with being less likely to recover at 1 year.
Wynberg et al. (13)	342 COVID-19 patients during the first 12 months after illness onset	Underweight or normal weight, BMI < 25 (140, 41%) Overweight, BMI: 25–30 (108, 32%) Obese, BMI > 30 (82, 24%)	In the 1-year post-COVID recovery study, the obese patients recovered 38% more slowly than participants with normal BMI (aHR 0.62, 95% CI = 0.39–0.97). Recovery was slower in those with a BMI ≥ 30 kg/m^2^ compared to BMI < 25 kg/m^2^ (HR 0.62, 95% CI = 0.39–0.97).
Xie et al. (99)	18,818 outpatients with COVID-19	Mean BMI: 27.64	In patients with COVID-19, obesity was independently associated with higher risk, with aHR of 1.83 (95% CI, 1.28–2.61).
Lacavalerie et al. (104)	51 chronic post-COVID-19 patients	Non-obese 18, Mean BMI: 25 Obese 33, Mean BMI: 34	Obese patients with chronic COVID-19 develop exaggerated ventilatory drive and impaired oxygenation at peak exercise, lower lung volumes, reduced ventilatory reserve (25 vs. 40, *p* = 0.011) and lower peripheral capillary oxygen saturation (96 vs. 98, *p* = 0.036).

COVID-19, coronavirus disease 2019; BMI, body mass index; OR, odds ratio; aHR, adjusted hazard ratio; aRRs, adjusted risk ratio; VTE, venous thrombus embolism; CI, confidence interval; HR, hazard ratio.

In addition, in acute COVID-19, higher BMI is associated with deep vein thrombosis and pulmonary embolism events. A study showed that after multivariate adjustment analysis, patients with Class II obesity (BMI 35.0–39.9 kg/m^2^) had a higher risk of thromboembolism than participants with normal BMI [hazard ratio (HR) 2.01, 95% CI 1.30–3.12] (9). Hypoxia, vascular endothelial injury, and thrombotic inflammation play a well-recognized role in exacerbating COVID-19 symptoms. The combination of insufficient cavity ventilation and poor pulmonary perfusion leads to severe respiratory distress symptoms (shortness of breath with the respiratory rate ≥ 30 times/min) or respiratory failure (19). Even with prompt mechanical ventilation, there can be a failure to reverse pulmonary conditions due to ventilation-perfusion mismatch (20) whereas maintaining normal oxygen saturation improves survival (21, 22). Biomarkers of vascular endothelial injury and platelet activation [such as von Willebrand factor (vWF) antigen, soluble E-selectin, soluble P-selectin, angiopoietin (Ang) 2, and soluble intercellular adhesion molecule-1] were maintained at high levels in hospitalized patients, and to a greater extent in ICU patients (23, 24). During the acute phase of COVID-19, pulmonary interstitial inflammatory infiltration and elevated levels of inflammatory markers (such as cytokines, chemokines, lactate dehydrogenase, C-reactive protein, ferritin, and procalcitonin) have also been reported in multiple imaging studies (25–27). Thromboembolic events accelerate the progression to severe disease in COVID-19, and even in recovering patients, microthrombi and pulmonary blood flow restriction have been reported (28, 29). Patients with obesity are more likely to exhibit vascular injury and develop hypoxemia and thrombotic inflammation during the acute phase of COVID-19. Therefore, obesity can aggravate of COVID-19 by affecting endothelial cells, inflammatory response, hypercoagulation, and thrombosis (30, 31). This review focuses on the relationship between hypoxemia, vascular endothelial injury, and thrombotic inflammation, the synergistic effect of obesity and acute COVID-19, the influence of obesity on long COVID, and suggestions for treatment.

## Hypoxia, vascular endothelial injury, and thrombotic inflammation in COVID-19

In one study of more than 900,000 COVID-19 patients followed for 90 days, the cumulative incidence of venous thromboembolism ranged from 0.2 to 0.8% and was as high as 4.5% in hospitalized cases (32). Another observational study involving more than 1 million COVID-19 cases showed that SARS-CoV-2 infection significantly increased the risk of thromboembolic events, with a 3-fold and 7-fold increase in the risk of deep vein thrombosis and pulmonary embolism, respectively, even in mild cases, although the risk was greater in severe cases. The heightened risk of venous thrombosis and pulmonary embolism lasted as long as 3–6 months (33). Damaged vascular endothelium is a crucial cofactor in forming *in situ* thrombosis. Biomarkers of endothelial activation and the formation of endothelial-derived extracellular vesicles were consistently observed in both acute and long COVID (23, 34, 35). Injured vascular endothelium exposes collagen, which binds to glycoprotein Ib/IX/V complex on the platelet membrane through the bridging molecule vWF to enhance platelet adhesion. The levels of protective factors (prostacyclin, nitric oxide, and NTPDase-1) decreased, weakening the inhibition of platelet activation, aggregation and expansion, thus forming a pro-thrombotic environment. Activated platelet phenotypes have been observed in convalescent patients after mild SARS-CoV-2 infection (36). Disruption of homeostasis leads to high intracellular Ca^2+^ concentration and the subsequent activation of Ca^2+^-dependent scramblase on the cell membrane. The resulting increased phosphatidylserine (PS) exposure on the outer membrane of injured vascular endothelial cells promotes activation of the intrinsic tenase complex and the formation of the prothrombinase complex (37, 38). The number of PS^+^ peripheral blood mononuclear cells in patients at the initial stage of COVID-19 diagnosis was higher than that of healthy controls (39). Moreover, tissue factor (TF) is also decrypted by PS, promoting the activation of the exogenous tenase complex. Anti-TF cannot completely inhibit the coagulation cascade, but lactadherin can inhibit coagulation in multiple ways by competing for PS binding sites (40). In the early stages of COVID-19, immune cells recognize and clear a small amount of virus without inducing severe inflammatory reactions, and laboratory tests show no significant changes or only decreases in peripheral blood white blood cell counts (41). In more severe cases, drastic replication and release of SARS-CoV-2 result in accumulation of immune cells in the lung tissue. Cytokines initiate the associated transduction pathways and trigger a cascade of inflammation that leads to cytokine storms (42–44). In addition, when immune cells remove pathogens, they release a large number of PS^+^ microvesicles. Thrombus and inflammation interact to further damage the vascular endothelium. Fogarty et al. recently reported that significantly elevated intermediate monocytes and activated CD4^+^ and CD8^+^ T cells were associated with sustained EC activation and poor hemostatic function in long COVID (45).

Vascular endothelial dysfunction and structural destruction also contribute to the development of hypoxemia. SARS-CoV-2 enters the alveolar interstitium and infects the capillary endothelial cells at the thin part of the air-blood barrier. The injured capillary endotheliocytes begin to contract and narrow the microcirculation. Moreover, due to the binding of SARS-CoV-2 and angiotensin converting enzyme 2 (ACE2), the available ACE2 is reduced, and the conversion of Ang II to Ang 1-7 is suppressed, causing vasoconstriction, inflammation promotion, enhanced vascular permeability, and pulmonary edema (46–48). ACE2 knockout mouse models exhibit more severe acute respiratory distress syndrome (49), and ACE2/Ang-(1-7) inhibition has been implicated in endothelial dysfunction or endotheliitis in COVID-19 stroke patients (50). The combination of damaged endothelial cells and vasoconstriction results in stenosis or obstruction in the tiny alveolar capillaries, interfering with normal gas exchange and promoting hypoxemia. Ackermann et al. found extensive alveolar capillary microthrombi, microangiopathy, and perivascular T-cell infiltration in the lungs of patients who died from COVID-19. The incidence of microvascular thrombosis in COVID-19 patients was 9 times higher than that in H1N1 patients (*p* < 0.001) and the number of new blood vessels was 2.7 times that of H1N1 patients (*p* < 0.001) (51). Neovascularization, capillary remodeling, microvascular sclerosis, and uneven vessel wall lead to the formation of local eddy currents, which promote platelet activation and PS exposure. Several studies have detected microclots in blood samples from long COVID patients and observed manifestations of poor blood flow in the vessels downstream of microthrombus-blocked capillaries (28, 29). At the same time, hypoxia can further damage the vascular endothelium through the release of free radicals, reactive oxygen species, and lipid hydroperoxide. The resulting decrease in adenosine triphosphate (ATP)-dependent translocation enzyme function influences membrane phospholipid stability and perturbs the normal function of endothelial cells.

Hypoxia and thrombosis can also be mutually aggravating, in addition to having common promoters. Hypoxia reduces ATP production, inhibits ATP-dependent translocase (flippase and floppase), and prevents the reversion of PS into the inner cell membrane. In addition to the vascular endothelial cells, various types of blood cells (such as red blood cells, platelets, neutrophils, lymphocytes, and monocytes) also exhibit abundant PS exposure, upregulating the coagulation cascade and accelerating thrombogenesis. PS is also exposed on the surface of the microvesicles released by these cells during apoptosis. The presence of pulmonary microcirculation thrombosis leads to pulmonary capillary hypertension, which increases the pressure difference between the two sides of the air-blood barrier. Driven by the pressure difference and the damaged alveolar structure, water molecules, albumin, and platelets enter the alveolar cavity, inducing increased blood viscosity and aggravated vascular stasis. In severe cases, macromolecules such as globulins and red blood cells also appear in the alveolar lumen. The increased fluid causes a decrease in the effective alveolar volume and exacerbates dyspnea and hypoxemia.

## Obesity and acute COVID-19

### Limited alveolar dilatation

In individuals with obesity (especially central obesity), adipose accumulation in the chest wall and abdomen restricts the lungs’ expansion and impedes diaphragm movement, resulting in a decrease in lung volume (52, 53). Visceral fat also increases airway resistance. Additionally, since alveolar ventilation and pulmonary blood flow progress from the apex to the bottom of the lung, compression of the lower part has more significant effects on lung function. Studies have shown that obesity itself can lead to respiratory impairment, with decreases in expiratory reserve capacity, functional capacity, forced vital capacity, functional residual capacity, expiratory reserve capacity, and total lung volume (2, 54).

### Chronic low-grade inflammation aggravates tissue damage and inflammation diffusion

**FIGURE 1:**
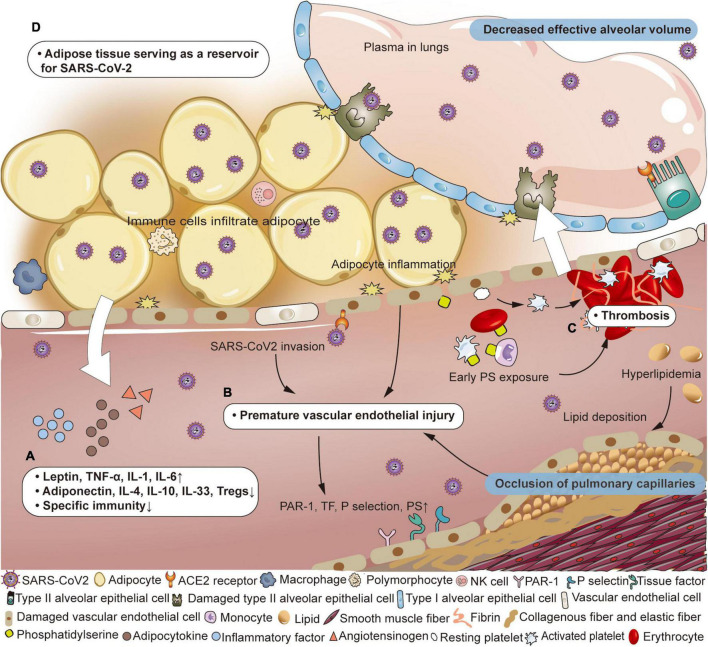
Pathophysiological changes of air-blood barrier in obese patients with COVID-19. **(A)** Adipose tissue has the potential to serve as a reservoir for SARS-CoV-2. **(B)** Immunocytes infiltrate adipose tissue, producing inflammatory mediators and adipokines, accompanied by a weakened specific immune response. **(C)** Under the combined effects of virus invasion, inflammation of adipocytes and lipid deposition, vascular endothelial destruction occurs prematurely, and the vascular wall structure is destroyed. The expression of protease activated receptor 1 (PAR-1), tissue factor (TF), P selectin, and phosphatidylserine (PS) on endothelial cells is up-regulated. **(D)** PS exposure on vascular endotheliocyte, erythrocyte, platelet, neutrophil, and lymphocyte appears earlier and participates in thrombosis. Adapted from “Adipocyte (white),” by BioRender.com (2022). Retrieved from https://app.biorender.com/biorender-templates.

Wherever SARS-CoV-2 goes, it recruits a variety of immune cells, and later inducing the release of cytokines, including monocyte chemoattractant protein 1, granulocyte-macrophage colony-stimulating factor, macrophage colony-stimulating factor, interleukin-1 (IL-1), tumor necrosis factor α (TNF-α), and IL-6 (55–60). As adipocytes swell to store excess energy, close interactions between adipocytes and host immune cells enhance lipolysis, resulting in abnormal adipocyte secretion (more leptin and less adiponectin), insulin resistance, and persistent low-level inflammation (61–63). Mitochondrial dysfunction and reactive oxygen species production induced by hyperglycemia promote vigorous generation of cytokines (such as TNF-α, IL-1, IL-6, IL-18, and interferon γ) (64, 65). In addition to the increase in pro-inflammatory mediators, anti-inflammatory regulatory substances (such as adiponectin, IL-4, IL-10, IL-33, and Tregs) are reduced. In terms of host defense, obesity suppresses the adaptive immune system against the influenza virus, suggesting that this could also be true for COVID-19 (Figure 1A; 1, 66). Although the immune system actively clears the pathogens, it does not produce completely specific anti-viral immune response, increasing the likelihood of viral escape. The abnormal obese state throughout the body promotes the inflammatory cascade, endothelial dysfunction and thrombosis, which worsens local tissue damage and facilitates the distant spread of SARS-CoV-2 (62, 67–69). Some scholars have proposed that the imbalance of the intestinal microenvironment and host immune system may mediate infection susceptibility in obese individuals (70).

### Endothelial dysfunction induces a pre-existing damaged and prothrombotic state

The primary function of vascular endothelial cells is to ensure unobstructed blood flow and maintain a barrier between the circulatory system and surrounding tissues. However, in patients with obesity, the level of adiponectin and nitric oxide is too low to effectively maintain this ordinary protective function. Because of obesity-related chronic inflammation, immunocytes infiltrate adipose tissue and release inflammatory factors, exerting adverse effects on peripheral vascular endothelial cells (5). Additionally, the high incidence of hyperlipidemia (increased low-density lipoprotein, triglyceride, and cholesterol) results in atherosclerotic plaque formation that renders the underlying vascular endothelium more susceptible to damage (46). Overall, although individuals with obesity have relatively mild endothelial dysfunction which rarely causes severe adverse effects, this impaired protective effect leaves them more susceptible to developing severe complications from other diseases. Under the dual impact of obesity and SARS-CoV-2, viruses can more easily invade vascular endotheliocytes due to early phase weakening of the air-blood barrier. As the disease progresses to the middle stages, endothelial dysfunction becomes prominent, leading to more vulnerable and rigid pulmonary vessels (Figure 1B). Hypoxemia is further exacerbated by pulmonary edema caused by the enhanced permeability of the alveolar membrane. Although endothelial change is not specific, endothelium-induced thrombosis plays a significant role in COVID-19 (71). Vascular endothelium expresses more protease activated receptor 1 (PAR-1), TF, P selectin, and membrane PS, and releases microvesicles, vWF, and clotting factor VIII (72). This alteration, together with increased soluble thrombomodulin and the surface chemokines, causes platelet overactivation and thrombosis (73).

### Enhanced procoagulant activity blocks vascular perfusion

After adjustment for age, sex, and race/ethnicity, class II obesity (BMI 35.0–39.9 kg/m^2^) was associated with a higher risk of thromboembolism compared with normal BMI (HR 2.01, 95% CI 1.30–3.12) (9). A retrospective multicenter cohort study showed that the BMI of COVID-19 patients with venous thrombus embolism (VTE) (26.9 vs. 23.2 kg/m^2^, *p* = 0.04) was higher than that of patients without VTE (10). Autopsy results showed fibrin deposition and thrombosis in both macro and micro pulmonary vessels. In patients with obesity, increased levels of fibrinogen, vWF and plasminogen activator inhibitor-1 causes hypercoagulability (66, 74). Endothelial cell dysfunction appears prematurely under the effect of high inflammation promoted by both obesity and SARS-CoV-2, further inducing PS exposure on the outer layer of endotheliocytes and forming microthrombi (Figure 1C). It has been reported that most of the PS^+^ microvesicles in COVID-19 patients are from the endothelium and platelets (75). Althaus et al. found higher levels of PS exposure on platelets from ICU patients with SARS-CoV-2 infection than in the non-severe group, and that PS exposure was associated with organ failure and elevated D-dimer (76). Because of the high viscosity and slow blood flow in patients with obesity, platelets are more likely to adhere to vascular endothelial cells and participate in coagulation function. In addition, the surrounding inflammatory state caused by the infiltration of immune cells into adipocytes damages the vascular endothelium, increasing the prevalence of procoagulant platelets, leading to thrombin formation. More importantly, these microthrombi can contribute to extrapulmonary thrombosis, leading to ischemia and necrosis of the corresponding organs (77). In addition, excess fat tissue produces high levels of angiotensin, which is rapidly converted into Ang II. Ang II accumulation changes local hemodynamics primarily through pulmonary vasoconstriction, constituting a pre-thrombus environment (78). In the later stage, fibrinolysis is inhibited by the depletion of fibrinolytic factors, decreasing the clearance of cross-linked fibrin and thrombi (79, 80). As a result of early blocked pulmonary blood perfusion, ineffective luminal ventilation occurs even while the alveolar structure is undamaged. Later, after diffuse alveolar injury, there is a lower proportion of air-blood exchange.

## Obesity and long COVID

### Viral persistence

Adipose tissue has the potential to serve as a reservoir for viruses (Figure 1D). Damouche et al. detected replicative human immunodeficiency virus (HIV) in adipose CD4^+^ T cells in six patients with antiretroviral therapy-controlled HIV, which contributing to viral persistence and long-term immune activation (81, 82). Another study in mice infected with H5N1 has found high virus titers in adipose tissue, including tissue attached to thymus, spleen, kidney, and heart (83). Evidence of SARS-CoV-2 infecting adipose tissue has also been found in patients with COVID-19. Martínez-Colón et al. have detected SARS-CoV-2 in adipose tissue around the heart and intestines of patients who died from COVID-19. *In vitro* experiments in which adipose tissue was cultured with SARS-CoV-2-containing solution showed that the virus infected and replicated within adipocytes (84). It is controversial whether adipose tissue mediates SARS-CoV-2 infection through the high expression of ACE2 (85, 86). Some studies have proposed that ACE2 RNA can occasionally be detected in fresh mature adipocytes, although no ACE2 protein is detected, suggesting that there may be other ways to mediate viral invasion (84, 87, 88). However, the detrimental effects of obesity on recovery from COVID are widely recognized. Overweight/obesity was associated with increased odds of symptoms lasting for 4+ weeks in longitudinal studies [odds ratio (OR) 1.24, 95% CI 1.01–1.53], and long COVID code in electronic health records (OR 1.31, 95% CI 1.21–1.42) (89). A retrospective matched-cohort study showed that patients with BMI > 30 kg/m^2^ had a 10% relative increased risk (aHR 1.10, 95% CI 1.07–1.14) of reporting prolonged symptoms compared to patients with a normal BMI (11). The PHOSP-COVID Collaborative Group found that obesity (patients with BMI > 30 kg/m^2^ vs. BMI < 30 kg/m^2^) is an independent factor associated with not feeling fully recovered 1 year after hospital discharge in both severe (70.8 vs. 29.2%) and very severe recovery clusters (64.0 vs. 36.0%) (12). In the 1-year post-COVID recovery study, the patients with obesity recovered 38% more slowly than participants with normal BMI, when controlling for the effects of age, sex, and comorbidities (aHR 0.62, 95% CI 0.39–0.97) (13). These finding raise the possibilities of exploring the specificity of long COVID symptoms in patients with obesity. More research is needed into whether adipose tissue provides a reservoir for the virus to re-emerge from during long COVID.

### Long-term inflammation

The cytokine storm induced during the acute phase by SARS-CoV-2 can develop into long-term systemic inflammation (65, 90, 91). At autopsy, infection-driving inflammation was found in almost all SARS-CoV-2-infected adipose tissue samples (84). The virus infects the immune cells in the adipose tissue and recruits a large number of inflammatory mediators, affecting the surrounding normal cells and causing inflammation to spread. Studies of high-resolution computed tomography lung scans of individuals recovering from COVID 6–12 months after discharge commonly showed ground-glass opacity (GGO) associated with pulmonary inflammatory exudation (92, 93). A prospective observational study demonstrated that higher C-reactive protein concentration was related to the more severe post-hospital cohort, and IL-6 was significantly increased in the moderate disease cluster compared with the mild cohort. Systemic inflammatory characteristics (e.g., serum C-reactive protein concentration > 5 mg/L) have no overall change between 5 months and 1 year (81.1 vs. 79.7%) (12) post-infection. In HIV infection, adipose tissue is thought to be a contributing factor to chronic immune activation/inflammation, with macrophages and CD4^+^ and CD8^+^ T cells in adipose tissue showing intense activation characteristics (81). Adipocytes and immune cells, acting as inflammatory partners, are also likely to promote and perpetuate persistent inflammation in long COVID (94–96). Moreover, the vascular endothelium of patients with obesity has a high risk of injury due to lipid deposition, and the presence of microthrombi stimulates the vascular wall, facilitating the formation of aseptic inflammation.

### Microclots

Although difficult to detect, the formation of microthombi can have a severe detrimental effect on microcirculation and can contribute to organ dysfunction such as impairment of respiratory function and renal function injury. Pretorius et al. detected fibrin amyloid microclots and activated platelets in blood samples from all 80 enrolled long COVID individuals by fluorescence microscopy (28). A prospective cohort study that analyzed hematological data from patients discharged from hospital with COVID-19 showed a decrease in mean D-dimer at 60 days of discharge compared with admission, but still elevated above baseline (900.71 vs. 1,350) (97). Townsend et al. evaluated coagulation markers in patients 4 months after initial COVID-19 diagnosis and statistically showed that 25.3% of patients had elevated D-dimer levels (>500 ng/ml), although prothrombin time and activated partial thromboplastin time returned to normal in >90% of patients (98). However, in many cases, microthrombus cannot be ruled out even if coagulation and thrombus-related indicators are normal. It has been reported that, while the results of blood tests and lung X-rays were basically normal, SPECT/CT of discharged patients indicated poor pulmonary blood flow caused by microthrombi blocking the microcirculation (29). Microclots are not easy to be detected. Often, when laboratory and imaging results are abnormal, microclots have affected organ function and need to be removed quickly. Therefore, it is necessary to focus on the indicators that may be related to the prothrombotic state to prevent the formation of microthrombi. Several studies have shown increased levels of vascular endothelial activation markers in long COVID (such as vWF antigen, vWF propeptide, and soluble thrombomodulin). *In vitro* studies have shown that convalescent plasma can damage vascular endothelial cells suggesting that the vascular endothelial cells can be continuously activated and in a pro-thrombotic state (34, 35). McCafferty et al. consistently observed that in samples from patients both in the acute and long COVID contained platelets with an activated phenotype (expressing activation markers CD62P and PAC1) (36). High PS level is also associated with poor prognosis in the convalescent period. It is reported that the levels of PS^+^ microvesicles^+^ peripheral blood mononuclear cells in the blood of patients with long COVID-19 are higher than those in the healthy cohort (39). Higher BMI was associated with deep vein thrombosis and pulmonary embolism, and Xie et al. showed that individuals with obesity were at higher clinical risk for post-COVID-19 VTE events than non-obese patients (aHR 1.83, 95% CI 1.28–2.61) (99).

### Hypoxia

Cardiopulmonary response to exercise may remain limited after hospital discharge in COVID-19 patients with obesity. Under the influence of airflow, fluid in the alveoli evaporates, leaving plasma proteins to form hyaline membranes, which can eventually develop into lung fibrosis and consolidation (20, 100). Studies have shown that convalescent patients still have symptoms of dyspnea, radiographic findings of interstitial lung infiltration and GGO, and impaired lung function at 1 year after discharge. In these individuals, the proportion of the diffusing capacity of the lung for carbon monoxide < 80% is associated with disease severity (92, 93, 101–103). Patients admitted with nasal catheters or mechanical ventilation were more likely to have diffusion disorders after discharge than patients who did not require supplemental oxygen (OR 4.60) (93). Meanwhile, obesity can have additional effects on the pulmonary sequelae in long COVID. Analysis of an observational study found that patients with obesity and chronic COVID-19 displayed exaggerated ventilatory drive and impaired oxygenation at peak exercise, accompanied by lower lung volume, decreased ventilation reserve (25 vs. 40, *p* = 0.011), and lower peripheral capillary oxygen saturation values (96 vs. 98, *p* = 0.036) 6 months after hospital discharge (104). The PHOSP-COVID Collaborative Group compared patient-reported outcomes between 5 months and 1 year and demonstrated that FEV1% < 80% predicted only minimal change, while cognitive impairment significantly improved (12). Compared with non-COVID-19 participants with obesity, post-COVID patients with obesity had significantly reduced oxygen pulse (66 vs. 76, *p* = 0.003), indicating poor cardiac function in convalescence (104). Both hypoxia and thrombotic inflammation can affect myocardial metabolism, resulting in loss of normal systolic and diastolic functions of the damaged myocardium, pulmonary congestion, and insufficient systemic circulation blood volume. Furthermore, due to the reduced effective lung volume, the blood in the pulmonary microcirculation cannot adequately carry oxygen, inducing reduced oxygen saturation. These effects may persist into the recovery period.

## Therapy

### Respiratory support

Hendren et al. showed that COVID-19 participants who were overweight and class I to III obese had a higher risk of requiring mechanical ventilation after multivariate analysis (OR 1.28, 1.54, 1.88, and 2.08, respectively) (9). In a Seattle study of 105 hospitalized patients with COVID-19, the survival rate was 98.9% in patients with oxygen saturation greater than 90%, while only 35 in 51 (68.63%) patients with arterial oxygen saturation < 90% survived. Ensuring sufficient blood oxygen saturation can effectively improve the survival rate of patients (21, 22). However, ventilation does not remove the etiological factors or produce lung healing; it merely keeps patients alive until their biological mechanisms can overcome SARS-CoV-2 (101). Many COVID-19 patients have almost no difficulty breathing until arterial oxygen partial pressure drops below 60 mmHg. The resulting dyspnea and shortness of breath are common symptoms which prompt patients’ initial visit, and thus the patient’s condition is often remarkably advanced (105–107). With further progression, pulmonary hyaline membrane or lung consolidation complicate gas exchange even with ventilation support (108). Therefore, even patients with mild symptoms should receive oxygen inhalation through a nasal catheter at 5 L/min upon admission, to maintain at least 95% peripheral oxygen saturation (21, 109, 110). In more serious cases, oxygen storage mask (initial flow 8–15 L/min) should be utilized and if there is no improvement after 1–2 h, progressive treatments [such as High-flow nasal cannula oxygen therapy, non-invasive ventilation, invasive ventilation, and extracorporeal membrane oxygenation (ECMO)] should be performed (111). In patients with obesity, particular attention should be paid to suitable methods (ECMO is contraindicated in patients with BMI > 45 kg/m^2^) and the risk of iatrogenic infection due to mechanical ventilation. Without the moisture and temperature regulation effects of nasal mucosa, inhaled gas can exert intense irritation to the airway, making it prone to infection. Nursing difficulties and persistent inflammatory state can both contribute to increased susceptibility to associated infection.

### Comprehensive antithrombotic therapy

A study involving 176,137 hospitalized COVID-19 cases found a difference in case fatality between patients with and without pulmonary embolism (28.7 vs. 17.7%) (112). In addition to directly affecting microcirculation, thrombosis can also lead to increased local intravascular pressure, aggravated pulmonary exudation, and pulmonary dysfunction. Severe hypoxemia and pulmonary hypertension can even lead to myocardial dysfunction. Therefore, it is possible to prevent disease progression if comprehensive treatment can be taken early in the disease to relieve the hypercoagulable state (including antiplatelet and anticoagulation), and if necessary to dissolve (micro) thrombi (113, 114). Early comprehensive antithrombotic therapies aim to maintain unobstructed blood flow and ensure adequate alveolar blood perfusion, without resulting in pulmonary arterial hypertension. It also seeks to delay the onset of pulmonary edema, thereby reducing the incidence of respiratory distress and respiratory failure. Unobstructed blood circulation also promotes the clearance of viruses and damaged blood cells, thus inhibiting the spread of inflammatory reactions and preventing disease progression. When the damaged vascular endothelium results in the exposure of basement membrane collagen, platelets react to the endothelium injury, interweave with fibrin, and become the starting point of thrombus formation. Inhibition of platelet adhesion, aggregation, and release can also be used as therapeutic targets, through the use of aspirin (75–100 mg/d), clopidogrel (75 mg, qd), and dipyridamole (100 mg, tid), thereby inhibiting the formation of intrinsic and extrinsic tenase and prothrombinase complexed caused by PS exposure on outer cell membranes (115). Santoro et al. found that in-hospital use of antiplatelet drugs was associated with lower mortality after multivariate adjustment [relative risk (RR) 0.39, 95% CI 0.32–0.48, *p* < 0.01] (116). Anticoagulant drugs, which are the most commonly used and studied drugs in clinical practice, can prevent thrombosis by inhibiting coagulation factors and activating antithrombin III. Results from multiple randomized controlled trials have shown that the use of therapeutic heparin in non-critically hospitalized COVID-19 patients reduces the number of days requiring organ support, the incidence of VTE and 28-day mortality events, and the proportion requiring respiratory support or IMV, compared with standard-dose thromboprophylaxis cohorts (117–120). However, a similar meta-analysis of non-critically ill patients with COVID-19 found that therapeutic thromboprophylaxis had a higher incidence of bleeding than standard-dose anticoagulation, including major bleeding (HR 1.86, 95% CI 1.04–3.33) and minor bleeding (HR 5.23, 95% CI 1.54–17.77) (121). Using thrombotic events (arterial and/or venous) as the primary endpoint, Spyropoulos et al. found a benefit of therapeutic anticoagulation in non-critically ill patients with elevated D-dimer (RR 0.46, 95% CI 0.27–0.81) (119). High D-dimer levels can predict poor prognosis of COVID-19. D-dimer levels have been found to be generally higher in critically ill patients than in mild patients (2.4 vs. 0.5 mg/L) (122). Standard doses of thromboprophylaxis are recommended for adults who are critically ill during hospitalization (123). Although guidelines for clinical inpatients recommend only standard or therapeutic doses, intermediate doses (defined as low molecular weight heparin bid or increased weight-based dosing that is less than the recommended therapeutic dose) are often used in clinical trials (123, 124). Drug distribution and metabolic clearance in patients with obesity may necessitate adjustments to dosing. The clinical effect of moderately increasing anticoagulant dose still needs to be investigated by high-quality trials (78, 125). For patients with body weight > 90 kg or BMI ≥ 30 kg/m^2^, enoxaparin 30–40 mg bid or UFH 7500 IU bid/tid can be used as a prophylactic dose, and enoxaparin can be used at a therapeutic dose of 1mg/kg bid. With regard to treatment of outpatients with COVID-19, there is currently no evidence to support the routine use of antithrombotic agents such as aspirin, factor Xa inhibitors, or low molecular weight heparin for the prevention of arterial/venous thrombosis or COVID-19 progression, and several studies were stopped early due to lower-than-expected primary event rates (126). Currently, the omicron variant is milder in pathogenicity, with a lower hospitalization rate, lower mortality rate, and shorter duration of acute symptoms than the delta variant, but with rapid bronchial replication and high transmissibility (127, 128). In the new round of COVID-19 transmission led by omicron variant, in addition to high-risk groups such as the elderly and people with underlying diseases or immune deficiencies, individuals with obesity (BMI ≥ 30) are also more likely to develop critical illness than the general population. For those without anticoagulant contraindications, the time of anticoagulation should be seized. Timely and sufficient anticoagulant treatment can effectively relieve hypercoagulability, prevent the occurrence of symptomatic thrombotic events, and improve the prognosis of patients with obesity.

### Other treatments

Systemic corticosteroids are recommended for patients with severe and critical illness but should be used with caution in patients with diabetes or underlying immune deficiency (129). In a randomized, controlled clinical study, dexamethasone (6 mg per day for 10 days) reduced mortality in patients requiring oxygen support, both on IMV (29.3 vs. 41.4%; RR 0.64, 95% CI 0.51–0.81) and non-IMV (23.3 vs. 26.2%, RR 0.82, 95% CI 0.72–0.94) (130). Intravenous dexamethasone plus standard care increased the number of ventilator-free days within 28 days in patients with COVID-19 related acute respiratory distress syndrome (6.6 vs. 4.0, *p* = 0.04) (131). Tocilizumab (4–8 mg/kg IV, single dose) is also recommended as an anti-inflammatory drug in many guidelines and is usually recommended in patients with peripheral capillary oxygen saturation ≤ 94% on room air and CRP ≥ 75 mg/L. It inhibits IL-6 signaling by reducing the binding of soluble and membrane-bound receptors (sIL-6R and mIL-6R) of IL-6 to block T cell activation, plasmocyte immunoglobulin secretion, and macrophage activity (132–134). Results of a meta-analysis showed a 12% reduction in mortality in the tocilizumab group compared with a control group that did not receive tocilizumab (RR 0.27, 95% CI 0.12–0.59) (135). JAK inhibitors (such as baricitinib), which blocks the signaling of inflammatory and immune responses, is also used in severely ill patients with pneumonia and hypoxia (136). Other medications may also be considered. Remdisivir, an adenosine analog, binds to new strands of viral RNA and leads to premature termination of virus replication, improving recovery and reducing adverse events (137). Statins, which improve immune system function and fight inflammation and oxidative stress, could also be an option in the treatment of COVID-19 (138). Cytokine storm is associated with a higher risk of multiple organ failure and death. During severe and critical episodes, anti-granulocyte-macrophage colony-stimulating factor and IL-6 inhibitors can reduce inflammation severity, clear cytokines, and reduce disease risk (139). While it is possible to target cytokine storms in theory, it does not play a significant role in clinical practice and can be used as a complementary therapy (64).

### Precaution

Patients with obesity are more likely to experience hypertension, hyperlipidemia and diabetes before admission, and the incidence of various comorbidities is high during their hospitalization. It is unlikely that even prompt treatment after hospital admission can normalize the rate of mechanical ventilation and the ICU occupancy in patients with obesity to the level of normal-weight patients. Therefore, for patients with obesity, daily weight management before COVID-19 diagnosis is critical in preventing COVID-19 and mitigating risk. A healthy diet and proper exercise help support immune health. As with everyone else, use of personal protective equipment and avoiding crowds are also important risk reducing strategies.

## Conclusion

The ongoing worldwide epidemic of COVID-19 is a problem that every country faces. Preventing the progression of COVID-19 to severe disease and reducing the incidence of sequelae are two major priorities for disease management. COVID-19 patients with obesity exhibit increased thrombotic inflammation and hypoxia, which are associated with mechanical compression, persistent inflammation, vascular endothelial damage, and hypercoagulable state. Obesity also contributes to the development and persistence of sequelae in long COVID, and may be involved in persistent viral presence, chronic inflammation, microclots, and hypoxemia, although reliable evidence from larger, high-quality studies is still needed. Although vaccines are important in preventing severe disease, effective treatment of COVID-19 is still critical, given continued viral mutation and the limited effectiveness of vaccines. Early intervention, including timely oxygen supplementation, prevention of microthrombi, and relief of the spread and persistent effects of inflammation can prevent or reverse disease progression and reduce the occurrence of sequelae.

## Author contributions

MX wrote the manuscript and drew the figure and table. XW and HJ searched the manuscript and provided comments. JS proposed the project, designed the study, and revised the structure. VN reviewed the text and polished the language. All authors read and approved the final manuscript.
